# A significant presence in atherosclerotic cardiovascular disease: Remnant cholesterol: A review

**DOI:** 10.1097/MD.0000000000038754

**Published:** 2024-07-05

**Authors:** Li Wang, Qingmei Zhang, Zhiyang Wu, Xiwei Huang

**Affiliations:** a Department of Cardiology, Quanzhou Traditional Chinese Medicine Hospital, Quanzhou, Fujian Province, China; b Department of Pediatrics, Quanzhou First Hospital, Quanzhou, Fujian Province, China; c Department of Emergency Medicine, Puning People’s Hospital, Jieyang City, Guangdong Province, China.

**Keywords:** atherosclerotic cardiovascular disease, remnant cholesterol, residual cardiovascular risk, triglyceride-rich lipoprotein

## Abstract

The current first-line treatment for atherosclerotic cardiovascular disease (ASCVD) involves the reduction of a patient’s low-density lipoprotein cholesterol (LDL-C) levels through the use of lipid-lowering drugs. However, even when other risk factors such as hypertension and diabetes are effectively managed, there remains a residual cardiovascular risk in these patients despite achieving target LDL-C levels with statins and new lipid-lowering medications. This risk was previously believed to be associated with lipid components other than LDL, such as triglycerides. However, recent studies have unveiled the crucial role of remnant cholesterol (RC) in atherosclerosis, not just triglycerides. The metabolized product of triglyceride-rich lipoproteins is referred to as triglyceride-rich remnant lipoprotein particles, and its cholesterol component is known as RC. Numerous pieces of evidence from epidemiological investigations and genetic studies demonstrate that RC plays a significant role in predicting the incidence of ASCVD. As a novel marker for atherosclerosis prediction, when LDL-C is appropriately controlled, RC should be prioritized for attention and intervention among individuals at high risk of ASCVD. Therefore, reducing RC levels through the use of various lipid-lowering drugs may yield long-term benefits. Nevertheless, routine testing of RC in clinical practice remains controversial, necessitating further research on the treatment of elevated RC levels to evaluate the advantages of reducing RC in patients at high risk of ASCVD.

## 1. Introduction

Atherosclerotic cardiovascular disease (ASCVD) has emerged as a chronic global epidemic in recent years. Despite significant advancements in the prevention and treatment of this disease, it remains the leading cause of mortality worldwide. Dyslipidemia plays a pivotal role in the initiation and progression of ASCVD. Extensive clinical studies have consistently demonstrated the association between low-density lipoprotein cholesterol levels and the risk of developing ASCVD. International guidelines universally recommend statin therapy for both primary and secondary prevention of ASCVD,^[1]^ owing to its profound reduction in low-density lipoprotein cholesterol levels and subsequent substantial decrease in ASCVD risk.^[2]^ However, despite the use of statins or other lipid-lowering agents to achieve guideline-recommended levels of LDL-C in clinical practice, the risk of ASCVD persists. Moreover, hypertriglyceridemia is a prevalent dyslipidemia in clinical practice, particularly among ASCVD patients with diabetes, obesity, metabolic syndrome, fatty liver, and chronic kidney disease.^[3,4]^ Therefore, triglycerides (TG) and triglyceride-rich lipoproteins (TRLs) have been recognized as risk factors for ASCDV for several decades.^[5]^ Regrettably, recent clinical trials involving patients receiving statins have demonstrated no significant reduction in ASCVD events when utilizing triglyceride-lowering agents.

Recent evidence suggests that remnant cholesterol (RC), associated with the remnants of TRLs, not only plays a crucial role in the pathogenesis of ASCVD but also significantly amplifies the risk compared to triglycerides and TRLs. Therefore, this review was conducted by applying the corresponding search strategy across multiple databases: PubMed: (“Remnant cholesterol” OR “Triglyceride-rich lipoprotein”) AND (“Atherosclerotic cardiovascular disease” OR “atherosclerosis”) AND (“Residual cardiovascular risk” OR “cardiovascular residual risk”). Embase: (“remnant cholesterol”/exp OR “triglyceride-rich lipoprotein”/exp) AND (“atherosclerotic cardiovascular disease”/exp OR “atherosclerosis”/exp) AND (“residual cardiovascular risk”/exp OR “cardiovascular residual risk”/exp). Web of Science: TS=(“Remnant cholesterol” OR “Triglyceride-rich lipoprotein”) AND TS=(“Atherosclerotic cardiovascular disease” OR “atherosclerosis”) AND TS=(“Residual cardiovascular risk” OR “cardiovascular residual risk”). The aim was to retrieve relevant literature to clarify the concept of remnant cholesterol, comprehensively summarize existing epidemiological, genetic, and clinical evidence on elevated remnant cholesterol levels, its relationship with atherosclerotic cardiovascular disease risk, and provide clinical guidance for treatment.^[6]^

## 2. Comprehending remnant cholesterol: Definition, metabolism, clinical significance, measurement, and calculation

### 2.1. Definition, metabolism, clinical significance

**Figure 1.**
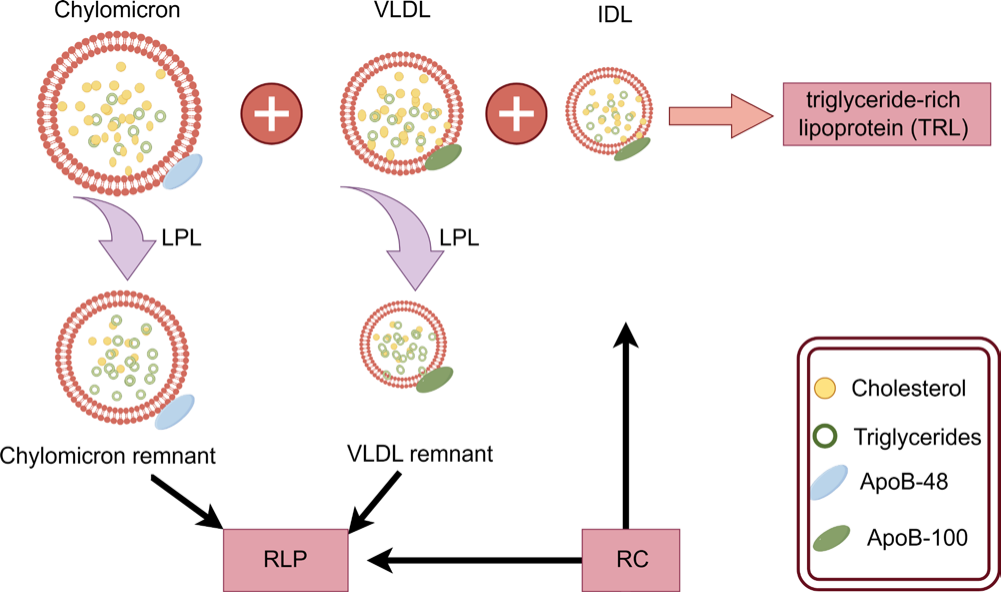
Triglyceride-rich lipoproteins include chylomicrons in the nonfasting state, VLDLs in the fasting state, and IDLs. Hydrolysis of chylomicrons and VLDL by lipoprotein lipase reduces triglyceride content, forming chylomicrons and VLDL remnants, respectively, which are also referred to as “RLP’’. The cholesterol component in IDL and RLP is called RC. This diagram has been created by the authors using Figdraw. IDL = intermediate-density lipoprotein, RLP = triglyceride-rich remnant lipoprotein particle, TG = triglyceride, VLDL = very low-density lipoprotein.

**Figure 2.**
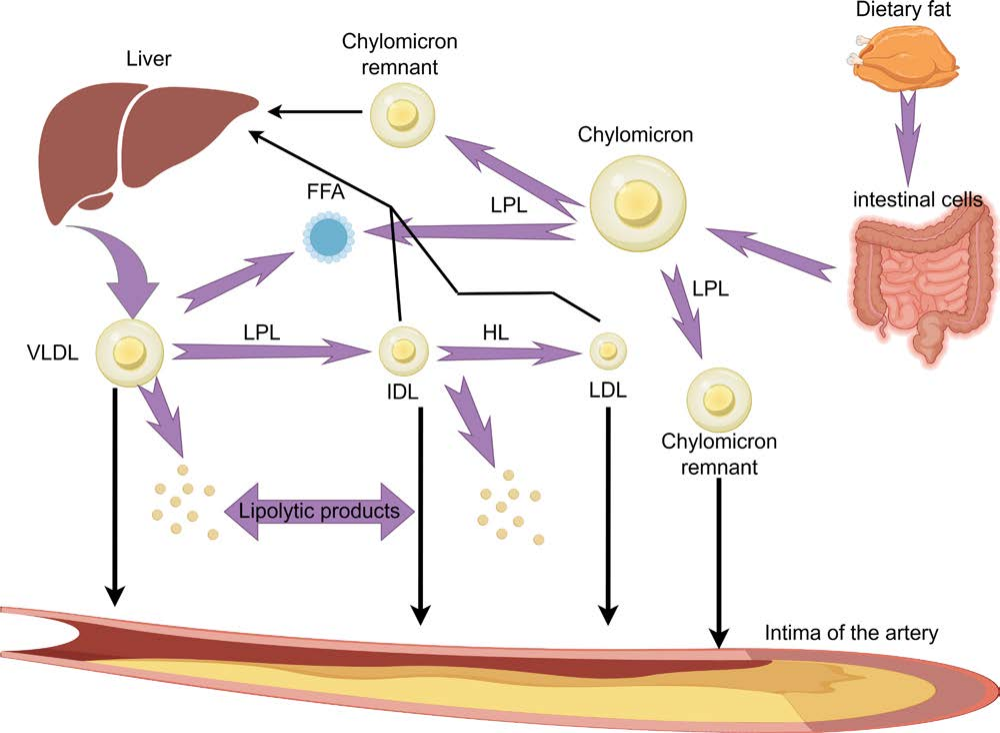
Endogenous pathway: when VLDL is secreted, it will bind various apolipoproteins such as apolipoprotein E, apolipoprotein C-I, apolipoprotein C-II, and apolipoprotein C-III, which will be hydrolyzed by LPL after entering the plasma to produce smaller VLDL particles, FFA, and IDL particles. IDL can be further lipolytic to LDL. VLDL particles, IDL particles, and LDL generated by the endogenous pathway can all penetrate the arterial intima. Exogenous pathway: after dietary fat is absorbed, TG is bound to CM. After CM enters the blood circulation, it is hydrolyzed by LPL to generate chylomicron residual particles and FFA. Residual chylomicron particles can also penetrate the arterial intima. This diagram has been created by the authors using Figdraw. CM = chylomicron IDL = intermediate-density lipoprotein, RLP = triglyceride-rich remnant lipoprotein particle, TG = triglyceride, VLDL = very low-density lipoprotein.

TG is the predominant constituent of adipose tissue in humans, and due to its hydrophobic nature, its transportation and metabolism within the human body necessitate the involvement of lipoproteins. TRLs, including chylomicrons (CMs) in the nonfasting state and very low-density lipoproteins (VLDLs) and intermediate-density lipoproteins (IDLs) in the fasting state, constitute core lipoproteins primarily composed of TG.^[7]^ RC represents the cholesterol component of TRLs (Fig. 1), encompassing VLDL and IDL during fasting and CM remnants during nonfasting states.^[8,9]^ Elevated plasma triglycerides are accompanied by increased TRL levels, which serve as a characteristic feature of elevated RC. These TRL originate from both endogenous pathways synthesized in the liver and exogenous pathways generated in the gastrointestinal system. The endogenous pathway primarily synthesizes VLDL in hepatocytes.^[10]^ As the apolipoprotein B100 scaffold, which contains phospholipids, triglycerides, and cholesteryl esters, undergoes progressive lipification, VLDL assembly takes place within the endoplasmic reticulum of hepatocytes. Additionally, VLDL is also generated in the endoplasmic reticulum of hepatocytes through the incorporation of free fatty acids (FFA) from circulation. During VLDL secretion process, various apolipoproteins such as apolipoprotein E, apolipoprotein C-I, apolipoprotein C-II, and apolipoprotein C-III bind to the surface of VLDL. When VLDL is secreted into the plasma, it undergoes hydrolysis by lipoprotein lipase (LPL) to generate smaller and denser VLDL particles, as well as FFA and IDL particles. The IDL particles can be taken up not only by residual receptors in the liver but also further subjected to lipolysis by hepatic lipase, resulting in the formation of LDL particles and lipolysis products. In the exogenous pathway, following absorption of ingested dietary fat by intestinal cells, TGs are bound to CMs containing apolipoprotein B48 (a truncated form of ApoB100). CMs are initially secreted into the intestinal lymphatic system and subsequently enter the bloodstream.^[11]^ Within the circulation, CMs acquire apolipoproteins such as apolipoprotein AI, apolipoprotein C-II, apolipoprotein C-III, and apolipoprotein E through high-density lipoproteins. Subsequently aided by apolipoprotein C-II, the CMs bind to LPL, leading to triglyceride hydrolysis and ultimately resulting in the production of residual CM particles and FFA. FFA can be regenerated into VLDL via the endogenous pathway or converted into glucose after β-oxidation by phosphoenolpyruvate carboxykinase. The glucose can then be reconverted back into triglycerides for storage in adipose tissue. However, liver clearance occurs for residual CM particles through binding with low-density lipoprotein receptor-like protein, low-density lipoprotein receptor, and heparan sulfate proteoglycan receptor. The metabolic pathways mentioned above reveal that VLDL in TRLs contains apolipoprotein B100 derived from the liver, while CMs contain B48 originating from the gut. It is widely believed that neither of these particles can induce atherosclerosis due to their inability to traverse the endothelium. Conversely, hydrolysis by LPL generates VLDL particles, IDL particles, and CM remnants which transport significant quantities of cholesterol molecules into the arterial intima, leading to a pronounced degree of atherosclerosis. Furthermore, owing to their extended residence within the vessel wall, remnants are considered to possess an atherogenic potential comparable to that of LDL-C^[12–14]^ (Fig. 2).

### 2.2. Measurement and calculation

RC refers to the cholesterol content in rlp, which poses significant challenges for accurate measurement due to the considerable heterogeneity in composition, size, mass, and density of various lipoproteins within rlp as well as their rapid metabolism. Currently, there are 2 main categories of methods used for measuring RC: the directly measured RC and the calculated RC. the directly measured encompass a range of detection techniques, primarily including direct automated determination, magnetic resonance spectroscopy, immunoseparation, and ultracentrifugation.^[15,16]^ Direct automated assays involve the use of enzymes and surfactants to eliminate cholesterol from other lipoproteins, followed by quantification of cholesterol in the remaining CM and VLDL. Magnetic resonance spectroscopy can also be employed to measure cholesterol in residual lipoprotein particles.^[17]^ The immunofractionation method was utilized to determine the cholesterol content of CM and VLDL remnants after removing apoA1 and ApoB100 using monoclonal antibodies against apo B-100 and ApoA1. Ultracentrifugation primarily measures the concentration of serum apolipoprotein 48 since the RC level from the exogenous pathway can be quantitatively evaluated based on the concentration of serum lipoprotein 48. However, regardless of which direct measurement method is used, it remains complex with relatively high costs associated with measurements; thus limiting its widespread application in clinical practice.^[18,19]^ The formula of total cholesterol-LDL-C-HDL-C is used to obtain the RC value.^[20,21]^ This method does not require special equipment and does not require additional cost, so it is more convenient in clinical use. It should be noted that when severe hypertriglyceridemia occurs, the calculation of LDL-C using the Friedewald formula^[22]^ is not reliable. Therefore, when TG is ≥400 mg/dL, the Martin-Hopkins equation^[23]^ is often used to calculate the LDL-C level more accurately, which is suitable for calculating RC in the case of higher TG.

In summary, given the limitations of all current methods for measuring RC levels, there is an urgent need to develop automated direct analytical methods for the accurate and convenient measurement of RC levels in plasma. In addition, due to the limitation of RC measurement methods, there is still no established normal range of RC, so determining the normal range of RC is also an urgent problem to be solved.

## 3. The role of remnant cholesterol in the pathogenesis of atherosclerotic heart disease: Epidemiology, genetic evidence, and atherogenic mechanisms

### 3.1. Epidemiology

The PREDIMED study by Castaner et al was conducted to examine the association between RC and major adverse cardiovascular events in people with a high risk of cardiovascular disease (overweight or obese). A total of 6901 participants were included in this study. For every 0.26 mmol/L increase in RC, the risk of cardiovascular events increased by 21%; when multivariable adjusted analyses were performed, RC levels, but not LDL-C levels, were associated with cardiovascular events in overweight or obese subjects. They also determined that a high risk of ASCVD was defined as an RC level ≥30mg/dL, regardless of whether or not the LDL-C level was within the target range of ≤100 mg/dL. In addition, many studies have confirmed the association between RC and cardiovascular disease in different ethnic groups.^[24]^ Studies from Japan have revealed the predictive effect of RC level on the recurrence of cardiovascular events in secondary prevention, and the use of RC values can be used for better stratification of patients’ cardiovascular risk. The study by Korean Huh et al showed that elevated RC levels (≥30 mg/dL) were associated with the incidence of cardiovascular disease in adults with type 2 diabetes, independent of LDL-C.^[25,26]^ The jackson Heart Study and the Framingham Offspring Cohort Study in the United States, representing different populations of black and white subjects, respectively, found no difference in the predictive effect of RC.^[27]^ Additionally, 3 prominent cohort studies conducted in the United States, namely the Atherosclerosis Risk in Communities Study, the Multi-Ethnic Atherosclerosis Study, and Coronary Risk Development in Young Adults, have demonstrated that elevated levels of RC are independently associated with ASCVD among individuals without preexisting cardiovascular disease,^[28]^ irrespective of traditional risk factors such as LDL-C, non-HDL-C, and apolipoprotein B levels.^[29]^

Although fasting lipid profiles are routinely used to assess cardiovascular risk, in the past few years, a number of prospective studies and clinical trials have confirmed that elevated nonfasting RC levels can predict an increased risk of the development and progression of atherosclerotic cardiovascular disease, and the predictive power is superior to that of LDL-C levels. In a study of 90,000 Danish subjects over 22 years, a progressive increase in nonfasting RC and LDL-C was associated with a similar increase in the risk of ischemic heart disease and myocardial infarction, but only the RC level was associated with all-cause mortality, and for every 1 mmol/L (39 mg/ dL) increase in RC, there was a significant increase in the risk of ischemic heart disease and myocardial infarction.^[6,16,30–32]^ The risk of coronary heart disease increases 2.8-fold. Another prospective study with 34 years of follow-up, the Copenhagen General Population Study (CGPS) and the Copenhagen Ischemic Heart Disease Study, which enrolled 73,513 subjects, confirmed the higher risk of nonfasting RC than LDL-C.^[6,17]^ For LDL-C level, the risk of ASCVD was found to increase by 10% for each 1 mmol/L increase in RC, whereas the risk of ASCVD increased by 40% for each 1 mmol/L increase in nonfasting RC level.^[32]^

### 3.2. Genetic evidence

The causal relationship between elevated RC and increased ASCVD risk was confirmed by Mendelian randomization studies. A comprehensive study of the CGPS, the Copenhagen City Heart Study, and the Copenhagen Ischemic Heart Study by Varbo A et al, which included 73,513 patients, Genes that increased RC and decreased HDL-C (tribles hololog1, glucokinase regulatory protein, and apolipoprotein A-V) and genes that decreased HDL-C only were analyzed. The results showed a causal risk estimate of 2.8 (95%CI, 1.9 to 4.2) per 1 mmol/L increase in RC level and 0.7 (0.4–1.4) per 1 mmol/L decrease in HDL-C. This indicates that increased RC and decreased HDL-C due to genetic variants are causally related to increased risk of ischemic heart disease, but variant genes that only lead to decreased HDL-C are not related to increased risk of ischemic heart disease.^[6]^ Nordestgaard et al analyzed 4 genetic mutations (APOA5, GCKR, LPL, and TRIB1) that specifically affect RC levels, compared with causal odds ratios and observed hazard ratios for patients exposed to different mutations, and concluded that for every 1 mmol per liter increase in RC, A 1.7-fold increase in the causal odds ratio and a 1.4-fold increase in the observed risk ratio were observed for myocardial infarction. The study by Tada et al pointed out that in the general population, the development and progression of ASCVD in patients with familial hypercholesterolemia are associated with serum RC levels.^[33]^ In addition, several recent Mendelian randomization studies of other variants in genes encoding apolipoprotein C-III, LPL, and angiopoietin-like proteins 3, 4, and 8 have also shown an increased risk of ASCVD with elevated RC levels.^[34–36]^

In conclusion, a robust causal association exists between elevated levels of RC and an increased risk of ASCVD, a conclusion substantiated by the current pertinent genetic studies.

### 3.3. Atherogenic mechanisms

In the process of the formation and development of atherosclerosis, RC participates in a key link through adhesion and proinflammatory effects, eventually leading to the occurrence of related diseases. In general, macrophages and smooth muscle cells, after progressively absorbing and accumulating RC across the vascular endothelium, transform into foam macrophages and eventually become part of atherosclerotic plaques.^[37,38]^ Once RC enters the intima and preferentially attaches to LDL-C through adhesion,^[19,29,39,40]^ reactive oxygen species production is increased by RC, with the result that vascular endothelial dysfunction is induced.^[41]^ Not only that, RC can act on the cell wall and increase the secretion of cytokines such as tumor necrosis factor-α and interleukin-1β,^[42,43]^ leading to the induction of apoptosis of endothelial cells, thereby promoting the formation of atherosclerosis.^[10]^ On the other hand, increased cytokine production can cause leukocyte migration and promote inflammation, which also leads to the formation of atherosclerosis. Not only that, but during RC hydrolysis by lipoprotein lipase, the release of FFAs and monoacylglycerol can lead to local inflammation, which is likewise involved in the formation and progression of atherosclerosis. Finally, RC is involved in the process of atherosclerosis by accelerating the assembly of thromboplastin complex, up-regulating the plasminogen activator inhibitor-1 gene, and expressing plasminogen activator inhibitor-1 antigen to enhance platelet activity and promote its aggregation, leading to the formation of thrombus or microthrombus.^[44–47]^

**Figure 3.**
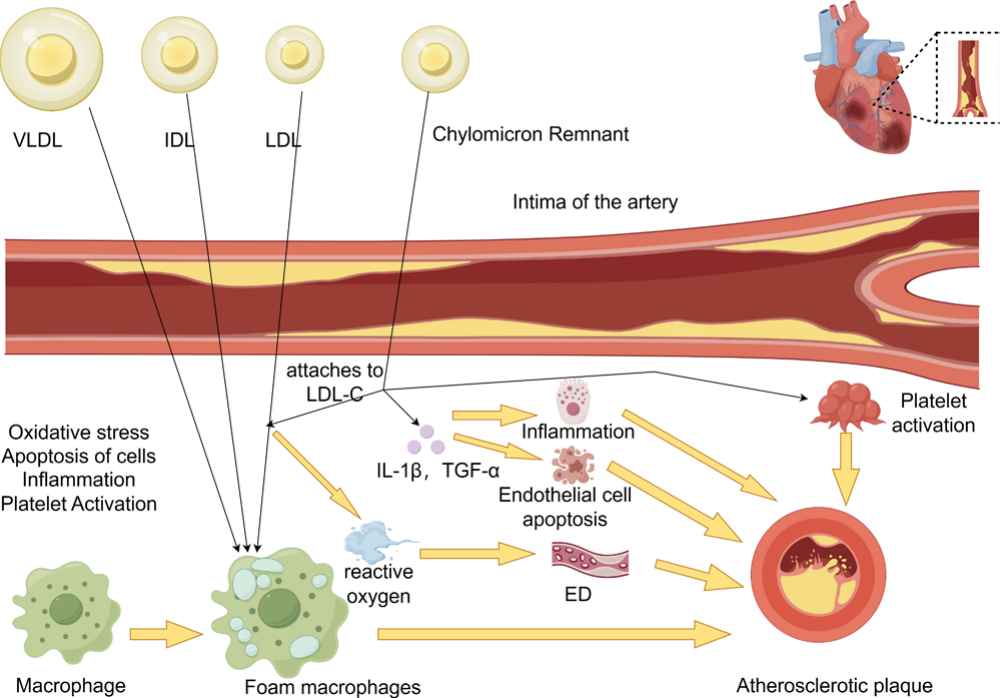
Cholesterol in the residue of TRLs metabolism, namely, RC, penetrates the intima of the artery, is absorbed by macrophages, and participates in the formation and development of atherosclerosis by promoting ED, causing inflammation, and accelerating thrombosis. This diagram has been created by the authors using Figdraw. ED = endothelial dysfunction, RC = remnant cholesterol, TRL = triglyceride-rich lipoprotein.

In conclusion, RC plays a pivotal role in the pathogenesis and progression of atherosclerosis by promoting endothelial dysfunction, eliciting and expediting inflammation, as well as instigating thrombosis (Fig. 3).

## 4. Treatment and management of elevated remnant cholesterol

Unhealthy lifestyle and poor dietary habits are the 2 most important aspects leading to dyslipidemia. Similarly, elevated RC levels are often associated with unhealthy lifestyles such as diets high in sugar or saturated fat, excessive alcohol consumption, overweight or obesity, and physical inactivity. Therefore, the management of elevated TRLs is focused on the intervention of unhealthy lifestyles, as recommended in the 2021 ESC guidelines.^[10]^ Healthy dietary practices include avoiding high carbohydrates, increasing the consumption of fiber-rich fruits and crops, consuming moderate amounts of seafood, limiting alcohol intake, and selecting for unsaturated fats.^[48]^ In general, by changing unhealthy lifestyle and maintaining healthy eating habits, RC level can be effectively reduced and cardiovascular disease can be prevented.^[12]^ Although the current guidelines still regard achieving LDL-C target values as the first priority for ASCVD patients, and do not directly recommend lowering plasma RC levels, there is no denying that more and more clinicians have realized that it is necessary to control other lipid components in ASCVD patients after achieving the recommended LDL-C levels. In order to achieve more benefits for ASCVD patients.

When it comes to using drugs to reduce remnant cholesterol (RC) levels, previous studies have indicated that commonly used clinical lipid-lowering drugs, such as statins, fibrates, omega-3 fatty acids (OM3FAs), and proprotein convertase subtilisin-kexin type 9 (PCSK9) inhibitors, all have a certain effect on reducing RC levels.^[24]^ A clinical study conducted in the United States demonstrated that statins can decrease RC levels in patients with atherosclerotic cardiovascular disease (ASCVD), with pitavastatin showing the strongest effect.^[49]^ Additionally, the ACCORD-LIPID study revealed that fenofibrate reduces the risk of ASCVD in patients with type 2 diabetes who are already receiving statin therapy. Similarly, research by Tsunoda et al suggested that fibrates, besides reducing triglycerides (TG) compared to placebo, also lower RC levels in patients with type 2 diabetes, indicating a potential reduction in ASCVD risk with fenofibrate due to its effect on RC levels.^[50,51]^ There is some controversy surrounding OM3FAs, as a meta-analysis involving 77,917 participants showed that while low-dose OM3FA supplementation reduced RC levels, it did not yield significant cardiovascular benefits.^[52]^ However, the REDUCE-IT trial^[53]^ reported contradictory findings. Nevertheless, it remains unclear whether the observed benefit was due to the combination of statins with OM3FAs or an increase in the daily dose of statins. In terms of PCSK9 inhibitors, a recent phase II trial demonstrated that PCSK9 inhibitors not only lowered LDL-C but also reduced RC by 42% to 52% compared with placebo.^[54]^

Current research also suggests that, in addition to conventional lipid-lowering drug treatments, new targeted lipid-lowering drugs based on genetic research may have potential effects on reducing RC levels. These include angiopoietin protein 4 antibodies, APOC3 inhibitors, APOB anti-therapeutic treatments, etc^[33,55–57]^ However, these new therapies targeting RC levels must undergo rigorous testing to prove their efficacy and benefits to patients, while ensuring they have no serious side effects, before being widely adopted in clinical practice.

## 5. Conclusion

In conclusion, the relationship between RC and cardiovascular disease is clear, and abnormal lipid metabolism is considered to be a major risk factor for ASCVD. RC plays an important role in predicting the incidence of ASCVD, which has been confirmed by epidemiological studies, genetic studies, and research on the pathogenic mechanism of RC. As a new indicator of atherosclerosis, RC has become a priority for treatment to reduce residual cardiovascular risk in persons at high risk for ASCVD, even when LDL-C levels are reduced to recommended levels. Although statins are still the cornerstone of lipid-lowering therapy, statins combined with other lipid-lowering drugs or new lipid-lowering drugs may bring more benefits to ASCVD and ASCVD high-risk patients when LDL-C is up to the target. However, the normal range of RC has not been determined, and there is no uniform measurement standard, and various detection methods are controversial. Therefore, more in-depth studies on RC detection are needed in the future, and more studies on the treatment of elevated RC and the cardiovascular benefits of ASCVD patients are urgently needed. Once these clinical benefits are confirmed, more patients at high risk of ASCVD will benefit in the future.

## Acknowledgments

We extend our thanks to all authors for their collaborative efforts. Special thanks go to my wife and all my family members for their silent support in my research work. All data presented in this article are publicly available, and sincere appreciation is extended to all experts who generously contributed to the research data presented in this article. It’s important to note that the researchers who provided the data to The Scientist do not necessarily endorse the comments made in this study. The author takes full responsibility for the content.

## Author contributions

**Conceptualization:** Xiwei Huang.

**Data curation:** Li Wang.

**Methodology:** Xiwei Huang.

**Resources:** Qingmei Zhang.

**Supervision:** Zhiyang Wu.

**Writing – review & editing:** Xiwei Huang.
